# Bacterial community dynamics and activity in relation to dissolved organic matter availability during sea-ice formation in a mesocosm experiment

**DOI:** 10.1002/mbo3.157

**Published:** 2014-01-20

**Authors:** Eeva Eronen-Rasimus, Hermanni Kaartokallio, Christina Lyra, Riitta Autio, Harri Kuosa, Gerhard S Dieckmann, David N Thomas

**Affiliations:** 1 Marine Research Centre, Finnish Environment Institute (SYKE)Erik Palménin aukio 1, PO Box 140, Helsinki 00251, Finland; 2 Department of Food and Environmental Sciences, University of HelsinkiPO Box 56, Viikinkaari 9, Helsinki, 00014, Finland; 3 Alfred Wegener InstituteAm Handelshafen 12, Bremerhaven, 27570, Germany; 4 School of Ocean Sciences, College of Natural Sciences, Bangor UniversityMenai Bridge, Anglesey, LL59 5AB, UK

**Keywords:** 16S rDNA, bacteria, DOM, flow cytometry, mesocosm, sea ice, T-RFLP

## Abstract

The structure of sea-ice bacterial communities is frequently different from that in seawater. Bacterial entrainment in sea ice has been studied with traditional microbiological, bacterial abundance, and bacterial production methods. However, the dynamics of the changes in bacterial communities during the transition from open water to frozen sea ice is largely unknown. Given previous evidence that the nutritional status of the parent water may affect bacterial communities during ice formation, bacterial succession was studied in under ice water and sea ice in two series of mesocosms: the first containing seawater from the North Sea and the second containing seawater enriched with algal-derived dissolved organic matter (DOM). The composition and dynamics of bacterial communities were investigated with terminal restriction fragment length polymorphism (T-RFLP), and cloning alongside bacterial production (thymidine and leucine uptake) and abundance measurements (measured by flow cytometry). Enriched and active sea-ice bacterial communities developed in ice formed in both unenriched and DOM-enriched seawater (0–6 days). *γ*-Proteobacteria dominated in the DOM-enriched samples, indicative of their capability for opportunistic growth in sea ice. The bacterial communities in the unenriched waters and ice consisted of the classes Flavobacteria, *α*-and *γ*-Proteobacteria, which are frequently found in natural sea ice in polar regions. Furthermore, the results indicate that seawater bacterial communities are able to adapt rapidly to sudden environmental changes when facing considerable physicochemical stress such as the changes in temperature, salinity, nutrient status, and organic matter supply during ice formation.

## Introduction

In addition to sea ice in the Arctic and Southern Oceans, seasonal sea ice also covers large areas of marine and brackish waters at lower latitudes, such as the Baltic Sea and the Sea of Okhotsk (Granskog et al. 2010). At its maximum extent, sea ice forms one of the largest ephemeral seasonal biomes on earth (Thomas and Dieckmann 2002). When sea ice forms, organic and inorganic components in the parent seawater are concentrated into brines that can collect in channels and pores before expulsion from the ice matrix due to a variety of largely physical processes (Petrich and Eicken 2010). At the same time, diverse assemblages of autotrophic and heterotrophic microorganisms can also colonize the ice matrix, living in interstitial spaces on and within the ice (Thomas and Dieckmann 2002; Arrigo et al. 2010; Caron and Gast 2010; Deming 2010).

Sea ice frequently contains high concentrations of dissolved organic matter (DOM, Thomas et al. 2010), which is often largely comprised of carbohydrate-rich algal-derived extracellular polymeric substances (EPS) produced by both bacteria and algae (Underwood et al. 2010, 2013; Krembs et al. 2011). However, it can also contain high concentrations of biologically labile compounds such as sugars and amino acids (Amon et al. 2001; Thomas et al. 2001). In Arctic and Baltic Sea ice, DOM has been shown to contain significant amounts of allochthonous components introduced from terrestrial systems through river inputs (Stedmon et al. 2007; Thomas et al. 2010).

Bacterial growth in sea ice is governed by the interaction of salinity, temperature, and nutrients (Pomeroy and Wiebe 2001; Kuosa and Kaartokallio 2006) as well as biotic factors such as grazing (Kaartokallio 2004; Riedel et al. 2007a). Bacteria seem to become entrained in sea ice along with phytoplankton (Grossmann and Gleitz 1993; Grossmann 1994; Grossmann and Dieckmann 1994; Helmke and Weyland 1995; Weissenberger and Grossmann 1998; Riedel et al. 2007b), whereas physical enrichment alone seems to be ineffective (Grossmann and Dieckmann 1994; Weissenberger and Grossmann 1998). Other possible explanations for bacterial entrainment in sea ice are gas vacuoles (Staley and Gosink 1999) and interaction with microgels in the EPS continuum (Ewert and Deming 2011). In newly formed sea ice, bacterial activity can be suppressed, but as the ice consolidates, bacterial activity has been shown to increase (Grossmann and Gleitz 1993; Grossmann 1994; Grossmann and Dieckmann 1994; Helmke and Weyland 1995; Kaartokallio 2004; Kaartokallio et al. 2008) and psychrophilic bacteria become more abundant (Helmke and Weyland 1995). There is evidence that the subsequent development of the bacterial communities is more dependent on the availability and lability of organic matter than on temperature (Helmke and Weyland 1995).

Bacterial succession in sea ice has been studied previously in the Baltic Sea and Arctic Ocean (Kaartokallio et al. 2008; Collins et al. 2010). In the brackish Baltic Sea, ice bacterial communities evidently changed throughout the winter (Kaartokallio et al. 2008). In the Arctic, sea-ice bacterial communities closely resembled seawater bacterial communities and therefore selection processes during freezing seem to play a relatively minor role at the clade level (Collins et al. 2010). These two studies focused on consolidated sea ice weeks after freezing. However, the development of bacterial communities in newly formed sea ice is unknown.

Sea ice from polar regions and the Baltic Sea, have similar bacterial communities at the class level, but their relative abundances vary depending on the location and time (Bowman et al. 1997; Staley and Gosink 1999; Brown and Bowman 2001; Junge et al. 2002; Brinkmeyer et al. 2003; Kaartokallio et al. 2008; Bowman et al. 2012). The main bacterial groups described from sea ice are from the phylum Bacteroidetes (e.g., class Flavobacteria), and the classes *α*-and *γ*-Proteobacteria, with less abundant groups such as *β*-Proteobacteria and Archaea also being reported (Deming 2010 and references therein). Frost flowers are ice crystals that form on young ice as a result of the upward expulsion of brine and cold but humid atmospheric conditions (Deming 2010 and references therein). Although the physical and chemical properties of frost flowers are relatively well characterized (Perovich and Richter-Menge 1994; Alvarez-Aviles et al. 2008), the biological components in frost flowers are not well-known (Bowman and Deming 2010; Bowman et al. 2013). Frost flowers have higher bacterial abundances, and bacterial communities significantly different, from those in the underlying sea ice (Bowman and Deming 2010; Bowman et al. 2013).

Certain bacterial groups are coupled with the quantity and quality of DOM in marine environments, under both natural and experimental conditions (Pinhassi and Berman 2003; Elifantz et al. 2005, 2007; Alonso-Saez and Gasol 2007; Mou et al. 2008; Teira et al. 2008, 2010, 2011; Alonso-Saez et al. 2012; Gomez-Consarnau et al. 2012; Teeling et al. 2012). For example, some *γ*-Proteobacteria are capable of opportunistic growth and are able to successfully exploit elevated concentrations of labile, low-molecular weight (LMW) compounds (Eilers et al. 2000; Fuchs et al. 2000; Pinhassi and Berman 2003; Allers et al. 2007; Teira et al. 2008, 2010; Gomez-Consarnau et al. 2012). In addition to *γ*-Proteobacteria, *α*-Proteobacteria can also effectively use LMW compounds (Cottrell and Kirchman 2000; Elifantz et al. 2005; Malmström et al. 2005; Alonso-Saez and Gasol 2007; Alonso-Saez et al. 2012; Teeling et al. 2012). Marine *α*-Proteobacteria, clade *Roseobacter* (family *Rhodobacteraceae*) thrive in nutrient-rich waters and is thought to metabolize exudates from growing phytoplankton (Allers et al. 2007; Teira et al. 2008; Alonso-Saez et al. 2012), whereas the often dominating *α*-Proteobacterial SAR11 clade is most competitive in oligotrophic waters (Morris et al. 2002; Malmström et al. 2005; Teira et al. 2010). Despite this knowledge about various bacterial groups and DOM associations, little is known about the relationships between DOM supply and bacterial communities in sea ice.

The aim of this study was to investigate the bacterial succession in sea ice as it formed and to evaluate the effect of the DOM content in the parent seawater on the developing sea-ice bacterial communities. An additional aim was to determine whether or not the sea-ice bacterial communities formed during freezing reflected parent seawater communities. The experiment was conducted in mesocosms in a large tank facility, using unfiltered seawater from the North Sea and the effect of DOM-enrichment was investigated, using algal-derived DOM.

## Experimental Procedures

### Sampling

The experiment was conducted at the Hamburg Ship Model Basin (HSVA) in Germany in October 2009. Water from the North Sea was collected near Helgoland (54°11′N, 7°55′E) and transferred with a clean food-quality road tanker to the HSVA within 24 h of initial collection. The water temperature was 13°C when collected and was maintained at this temperature (±1°C) during transport. Eighteen polyethylene (PE) bags were placed into the test basin, using a random block design, and each bag was filled with 1.2 m^3^ of North Sea water. After filling, the air temperature was set to 0° to cool the water. To keep the water well mixed during the experiment, each bag was also equipped with a simple circulation pump. To prevent autotrophic growth, the experiment was carried out in darkness, except during sampling (2–3 h per day), which was done at low light. Protists were counted by epifluorescent microscopy and they were found only in very low numbers (Aslam et al. 2012).

GF/F-filtered algal-derived organic matter (described in Aslam et al. 2012) was introduced to nine of the mesocosms, while the remaining mesocosms contained only North Sea water. The mean dissolved organic carbon (DOC) and dissolved organic nitrogen (DON) concentrations in the DOM mesocosms were 388.1 and 45.3 *μ*mol L^−1^ and in the NSW 109.4 and 4.18 *μ*mol L^−1^, respectively. The first sampling (day 0) was performed in all 18 mesocosms 2 days after water had been pumped into the mesocosms.

Subsequently, freezing of the water was initiated by decreasing the air temperature to −13°C (±2°C) and spraying a fine mist of Milli-Q water (EMD Millipore Corp., Billerica, MA) over the surface of the basin to ensure ice nucleation (Giannelli et al. 2001). To allow for water pressure equilibration in the under ice water and to ensure that the underside of the developing ice was always in contact with the underlying water, open-ended polyvinyl chloride (PVC) tubes were placed in a corner of each PE bag. Ice was removed from these tubes every day, thereby releasing pressure, after which the tubes were used for under ice water sampling.

Two days after freezing commenced (day 3), the sea ice had grown <1 cm and the ice was too thin and unstable to perform all the measurements needed. Therefore, the ice cover was left intact, and only the under ice water was sampled from all bags through the pressure-release PVC tubes. The sea ice had consolidated by day 4 and ice and underlying waters from three replicate mesocosms were sampled from both the DOM-enriched and-unenriched treatments on days 4–6. Unfortunately on the first ice-sampling day (day 4), one of three replicates of the DOM-enriched and two of three replicates of the unenriched mesocosms were compromised and not included in the subsequent analyses. Once a mesocosm had been sampled for ice it was not sampled again, as the removal of the ice completely altered future ice growth. Brine and frost flowers were collected on the last two sampling days (days 5 and 6).

Under ice water sampling was performed through the pressure-release PVC tubes, using 50-mL plastic syringes and small-bore Teflon tubes. Ice was sampled by sawing ice blocks, which were then floated in a container held beneath the ice to minimize brine drainage from the skeletal layer. The ice blocks were carefully removed and immediately cut into two or three sections depending on ice thickness: The top and middle ice sections were always 4 cm and the bottom ice varied between 3 and 8 cm. The ice sections were placed into autoclaved polypropylene (PP) buckets, and melted at room temperature (melt water not exceeding 4°C). As soon as the ice had melted the samples were transferred to a cold room (4°C) and the water was filtered within 2 h.

Brines were collected on days 5 and 6 from sackholes (Thomas et al. 2010) drilled to a depth of 6 cm, using a Cherepanov ice drill (Ø 20 cm, see Aslam et al. 2012). Frost flowers were collected on days 5 and 6, by carefully scraping them into 1-L autoclaved PP containers. The rim of the container was used as a scraper to minimize any possible contamination. Similar to the sea-ice, frost flowers were melted at room temperature (melt water not exceeding 4°C), transferred to the cold room (4°C), and filtered within 2 h. Direct melting was used to avoid nonspecific addition of DOM and/or DNA and dilution of the sample. Direct melting has been shown to be appropriate for both Antarctic and Baltic Sea ice bacterial samples (Helmke and Weyland 1995; Kaartokallio 2004).

For DNA extractions 50–600 mL of melted sea ice, frost flowers, brines, or under ice water were filtered through sterile 0.22-*μ*m membrane filters (Ø 47 mm; Whatman, GE Healthcare, Kent, UK). All filters were immediately frozen at −20°C and subsequently stored at −80°C. Organic and inorganic nutrients, carbohydrates, oxygen, temperature, and salinity data were also collected during the experiment. The methods employed are described by Aslam et al. (2012) and Müller et al. (2013).

### Bacterial abundance and cell parameters

Samples for the determination of bacterial abundance were fixed with 0.2 *μ*m filtered microscopy-grade glutaraldehyde (final concentration, 0.5%) and stored at 4°C. The cells were stained with SYBR Green I (Molecular Probes, Eugene, OR) at a final dilution of 1:10 000 for at least 10 min in the dark and analyzed with an LSR II flow cytometer (BD Biosciences, San Jose, CA) using a 488-nm laser (essentially after Gasol et al. 1999; Gasol and Del Giorgio 2000) within 30 min of staining.

CountBright beads (Molecular Probes) were added to each sample to calculate the volume of sample used in counting. Bacterial data were typically acquired until 50,000 events were recorded. Cell populations of high-and low-nucleic acid content (HNA and LNA, respectively) bacteria were identified from bivariate plots of green fluorescence versus SSC (sideward light scatter), based on differences in green fluorescence using FACS Diva software (BD Biosciences). The cell abundance (cells per mL) for each population was calculated from the sample volume and number of recorded events identified as bacteria.

Bacterial abundances were normalized to a salinity of 33, which was the average salinity in the initial water samples. Salinity normalization was performed to enable comparison of the sea-ice communities with the initial water samples (see discussion by Müller et al. 2013). The enrichment index for bacteria in sea ice was calculated with the formula *I*_S_ = (*X*_i_/*S*_i_) * (*S*_W_/*X*_W_) (Gradinger and Ikävalko 1998), where *I*_S_ is the enrichment index, *X*_i_ is the unnormalized bacterial abundance in ice, *X*_W_ the unnormalized bacterial abundance in water, *S*_i_ the salinity in ice, and *S*_W_ the salinity in water.

### Bacterial production

Bacterial production measurements were carried out to evaluate the bacterial net biomass production, based on amount of DNA and protein synthesis. The samples contained a known amount of crushed ice and sterile-filtered seawater and were processed with the following techniques, as described by Kaartokallio (2004): In short, each intact ice section was crushed, using a spike and electrical ice cube crusher. Approximately 10 mL of crushed ice was weighed in a scintillation vial. To better simulate the brine pocket salinity and ensure even distribution of labeled substrate, 2–4 mL of sterile (filtered through a 0.2-*μ*m polycarbonate filter) seawater from the sample bags was added to the scintillation vials. All the work was done at 4°C.

Bacterial production was measured immediately after sample collection, using ^14^C-leucine (Leu, Kirchman et al. 1985) and ^3^H-thymidine (TdR, Fuhrman and Azam 1980, 1982) incorporation methods with dual labeling: Two aliquots and a formaldehyde-killed absorption blank were amended with L-[U-^14^C] Leu (PerkinElmer, Waltham, MA, specific activity 318 mCi mmol^−1^) and [methyl-^3^H] TdR (PerkinElmer, specific activity 20 Ci mmol^−1^). The concentrations used, 30 nmol L^−1^ for TdR (all sample types) and 1200 nmol L^−1^ (ice samples) and 400 nmol L^−1^ (water and brine samples) for Leu, were verified to be above the saturating concentrations (cf. Kaartokallio et al. 2013). The samples were incubated in the dark at −0.5°C according to the predicted level of activity: The ice samples were incubated for 18–22 h, water samples for 4–5 h, and brine samples for 8–9 h. The incubations were stopped by adding formaldehyde, and the samples were processed, using the standard cold trichloroacetic acid (TCA) extraction procedure (Fuhrman and Azam 1980, 1982). A Wallac WinSpectral 1414 counter (Wallac Oy, Turku, Finland) and InstaGel (PerkinElmer) cocktail were used for scintillation counting.

The Leu and TdR incorporation rates were normalized to a salinity of 33, which was the average salinity in the initial water samples. Salinity normalization was performed to enable comparison of the sea-ice communities with the initial water samples.

### DNA extraction and PCR amplification

DNA was extracted from the filters, using a PowerSoil® DNA isolation kit (MoBio Laboratories, Inc., Carlsbad, CA) according to the manufacturer's instructions with one additional step: the filters were crushed with a sterile pipette tip after they were transferred to the PowerBead tubes. The extracted DNA was used as a template (10–150 ng) to amplify the 16S rRNA genes for the terminal restriction fragment length polymorphism (T-RFLP) and clone libraries. For T-RFLP, PCR was performed with a 6-carboxyfluorescein-labeled forward primer (FAM27f FAM-GAGTTTGATCMTGGCTCAG, Sait et al. 2003; HPLC-purified, Oligomer Oy, Helsinki, Finland) and unlabelled reverse primer (1406r ACGGGCGGTGTGTRC, Lane et al. 1985; HPLC-purified, Oligomer Oy), whereas for the clone libraries both primers were unlabelled. The PCR reactions and purifications were performed, as described by Sinkko et al. (2011), except that three parallel PCR reactions from each sample were performed in a 25-*μ*L reaction volume and DyNazyme™ EXT DNA polymerase was used (Finnzymes, Thermo Fisher Scientific, Vantaa, Finland).

### Fingerprinting of the bacterial community, cloning and identification of terminal restriction fragments

The bacterial community composition was determined with T-RFLP (Liu et al. 1997) and cloning. The digestions and T-RFLP were performed with three different restriction enzymes (BsuRI, MspI and RsaI; Fermentas, Thermo Fischer Scientific, Burlington, ON, Canada; work done in the Helsinki University, Institute of Biotechnology, Helsinki, Finland), as described in Sinkko et al. (2011). The true peaks were determined with the statistical method developed by Abdo et al. (2006). Fragments from 26.5 to 1000 base pairs (bp) were included in normalization with BsuRI and 49.5 to 1000 with MspI and RsaI.

In all, three clone libraries were constructed to identify terminal restriction fragments (T-RFs) obtained, one from unenriched seawater on day 0, another from unenriched bottom ice on day 5 and the third from DOM-enriched bottom ice on day 5. Cloning of the amplified 16S rRNA genes, plasmid extractions, and sequencing were performed in Helsinki University, Institute of Biotechnology. Approximately 950 bp of the 16S rRNA gene were sequenced from the 5′ terminus of the 16S rRNA gene. The sequences were corrected manually with the Staden Package 1.6.0 Gap v. 4.10 (Staden et al. 1998, 2003) and putative chimeras were checked with Bellerophon in Greengenes (http://greengenes.lbl.gov/). Taxonomic identification of the 16S rRNA genes was done, using a naïve Bayesian classifier (v. 2.4, RDP training set 7) of the Ribosomal Database Project (RDP, Wang et al. 2007) by applying an 80% confidence threshold. To determine the closest sequence matches, the sequences were blasted against an RDP database (release 10.28, v. 3) using the Seqmatch tool with default options, although NCBI taxonomy was used (Cole et al. 2009). The 16S rRNA gene sequences were deposited in EMBL Nucleotide Sequence Database under accession numbers from HE979561 to HE979715.

The sequencing effort was estimated by calculating the operational taxonomic units (OTUs), using the average neighbor algorithm and 10,000 iterations in Mothur v. 1.21.1 (Schloss et al. 2009). Representative OTUs and the Chao1 richness index were also calculated with Mothur, and species evenness was calculated using Simpson's index (1/*D* = 1/∑*p*_i_^2^). Mothur LIBSHUFF (Schloss et al. 2004) was performed to statistically compare the bacterial community structure between libraries. The chloroplast sequences (two sequences in clone library 1) were omitted from all clone library analyses, ensuring that only true bacterial sequences were used in the analyses.

The T-RFs were identified with in silico 16S rRNA clone library digestions using the Restriction Enzyme Database (REBASE 7.11,version 1.20080403) virtual digest program (http://insilico.ehu.es/restriction/main/, Roberts et al. 2010) and in vitro. For the in vitro analysis, the 16S rRNA clones were PCR-amplified, digested, and analyzed with T-RFLP from plasmids, as described above.

### Phylogenetic analysis of the 16S rRNA gene

The sequences were aligned, using RDP aligner (release 11, update 1, Cole et al. 2009) and a bootstrapped (1000) phylogenetic neighbor-joining (NJ) tree with the Jukes–Kantor evolution model was constructed from the 16S rRNA gene sequences (˜900 bp) using Phylip 3.695 (Felsenstein 2005). Sequence from archaeon *Sulfolobus tokodaii* (AB022438) was used as an outgroup in the alignment. A phylogenetic NJ tree was visualized with Interactive Tree Of Life (iTOL, Letunic and Bork 2007).

### Statistical analysis of bacterial communities

Differences in the environmental data as well as bacterial abundance and production parameters between treatments and over time were tested, using Wilcoxon rank sum tests with continuity correction (W) and the Kruskal–Wallis (KW) rank sum test, respectively, with a base package of R software (R Development Core Team 2011).

To visualize the bacterial community structure based on T-RF data, principal coordinate (PCO) analysis was performed on the Bray–Curtis distance matrix derived from square root transformed relative abundance data. Square root transformation was performed in order to balance rare and abundant species, as the Bray–Curtis resemblance measure uses no form of scaling and our samples showed large differences between their relative abundances.

Generalized discriminant analysis based on distance (Anderson and Robinson 2003) was performed to test whether the bacterial communities could be discriminated by DOM addition and/or time. To calculate the *P*-values, 9999 permutations were used. All community structural analyses were performed, using PRIMER v. 6 (Clarke and Gorley 2006) with the add-on package PERMANOVA+ (Anderson et al. 2008).

## Results

### Environmental parameters

The salinities and temperatures during the experiment are shown in Table 1. No statistical differences between the treatments were observed, either in water or ice salinity and temperature (W test with continuity correction). Based on time, there were no statistical differences in sea-ice salinity or temperature, whereas in water both salinity and temperature changed significantly (KW: 39.2805, df = 4, *P* = 6.096 × 10^−8^, KW: 56.2111, df = 4, *P* = 1.811 × 10^−11^, respectively). As there were no statistical differences, either in salinity or temperature in the ice, the differences in brine salinity were most likely caused by the sampling techniques used. The average ice thickness varied from day 4 to day 6 by 7.5 cm (±0.5 cm, *n* = 10), 10.3 cm (±1.5 cm, *n* = 16), and 11.2 cm (±2.3°C cm, *n* = 11), respectively.

**Table 1:** Salinity and temperature measurements from North Sea water and sea-ice experimental units.

	Temperature (°C)			Salinity		
	Average	Range	*n*	Average	Range	*n*
Day 1						
Open water	0.5	0.3–0.7	15	33	32.8–33.1	15
Day 3						
Under ice water	−1.7	−1.8 to −1.6	15	33.8	33.2–35.2	13
Day 4						
Brine	ND	ND		63.5	59–68	2
Top ice	−8	−8.2 to −7.8	3	10.9	9.9–11.8	3
Bottom ice	−2.5	−2.6 to −2.4	3	15	14.8–15.3	3
Under ice water	−1.8	−1.9 to −1.8	15	35.5	34.5–36.9	3
Day 5						
Frost flowers	ND	ND		33.2	28–38.5	6
Brine	−4.3	−5.1 to −3.1	6	76.9	71.2–81.2	6
Top ice	−6.8	−7.3 to −6	6	10.1	9.1–10.8	6
Bottom ice	−2.3	−2.4 to −2.3	6	12	11.2–13	6
Under ice water	−1.9	−1.9 to −1.8	12	35.7	35.1–36.6	6
Day 6						
Frost flowers	ND	ND		36.7	26–42	6
Brine	−5.5	−6 to −5.3	6	87.8	85.4–90.4	6
Top ice	−7.4	−7.6 to −7	6	10.3	9.6–11.5	6
Middle ice	−4	−4.7 to −3.7	6	9.4	9.1–9.6	4
Bottom ice	−2.5	−2.7 to −2.3	6	12.8	10.7–14.3	6
Under ice water	−1.9	0	6	37.3	36.1–38	6

*n* = number of data points; ND = not determined.

### Bacterial abundances

**Figure 1:**
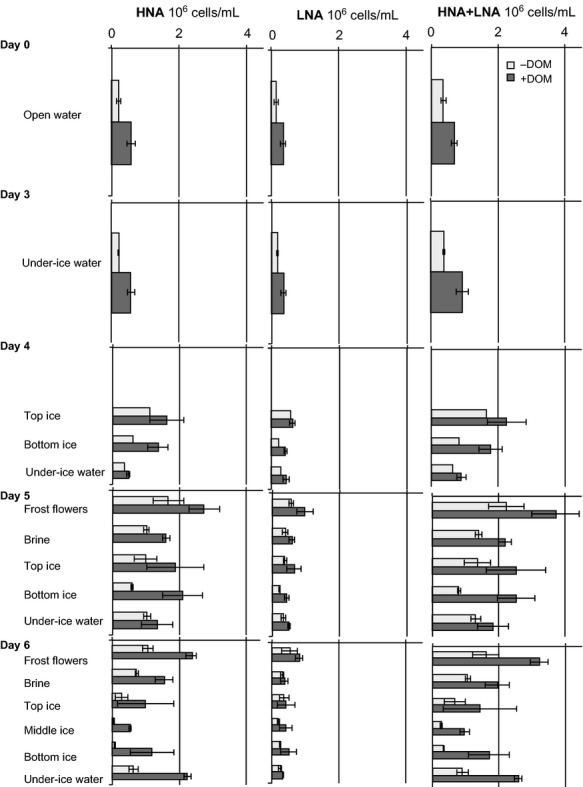
Abundance of flow cytometry-based bacterial cell populations in North Sea water and sea-ice experimental units. HNA refers to high-nucleic acid content and LNA to low-nucleic acid content. −DOM refers to unenriched and +DOM-enriched samples. Bars denote standard deviation between experimental units sampled on the respective sampling occasion.

The proportion of salinity normalized flow cytometry-based low-(LNA) and high-(HNA) nucleic acid content populations and total bacterial abundances (HNA + LNA) are presented in Figure 1. During the entire experiment, the total bacterial abundance, as well as the abundances of the HNA and LNA populations, were significantly higher in the DOM-enriched samples than in the unenriched samples in both ice (*W* = 275, *P* = 1.469 × 10^−5^; *W* = 267, *P* = 6.723 × 10^−5^; *W* = 259, *P* = 0.0002548, respectively) and water (*W* = 404, *P* = 5.02 × 10^−5^; *W* = 367, *P* = 0.00228; *W* = 452, *P* = 2.081 × 10^−8^, respectively). As the number of heterotrophic nanoflagellates was low throughout the experiment, as verified by epifluorescence microscopy, bacterial abundances give an estimate close to the maximum values attainable without flagellate grazing (see Aslam et al. 2012).

On the first ice-sampling day (4), the bacterial enrichment index (*I*_s_) (Gradinger and Ikävalko 1998) was 6.6 in the unenriched and 5.9 in the DOM-enriched samples, indicating a clear enrichment of bacteria in both treatments compared with the initial water samples (day 0). The highest bacterial abundances were measured in the frost flower samples. The HNA cells comprised a greater percentage of the populations in both the unenriched and DOM-enriched samples in all sample types. At the end of the experiment, the total bacterial abundance in both the unenriched and DOM-enriched ice samples significantly decreased (Fig. 1, *W* = 6, *P* = 0.01199; *W* = 7, *P* = 0.0293, respectively), whereas significant differences were not detected in the under ice waters.

We observed formation of bacterial cell aggregates (counted by epifluorescence microscopy) in both the unenriched and DOM-enriched samples on the last sampling day (day 6). In the DOM-enriched samples, the aggregates were found in water (*n* = 3), brine (*n* = 3) and ice (*n* = 1), whereas in the unenriched samples, only one ice sample contained small aggregates. In the DOM-enriched samples, the aggregates were estimated to contain on average 130–160 cells in brines and 200–240 cells in water, counting an average of 44% and 48% of all bacterial cells, respectively. In the unenriched samples, the aggregates were smaller, containing only 8–14 cells and constituting on average 11% of all bacteria. As the aggregates were detected only in one ice sample in the DOM-enriched samples and the proportion of the aggregates in the unenriched samples was very low, the bacterial cell aggregation observed cannot explain the declining abundance in the DOM-enriched ice samples. Instead, the aggregation may point out the onset of stationary growth in the experiment.

### Bacterial production

**Figure 2:**
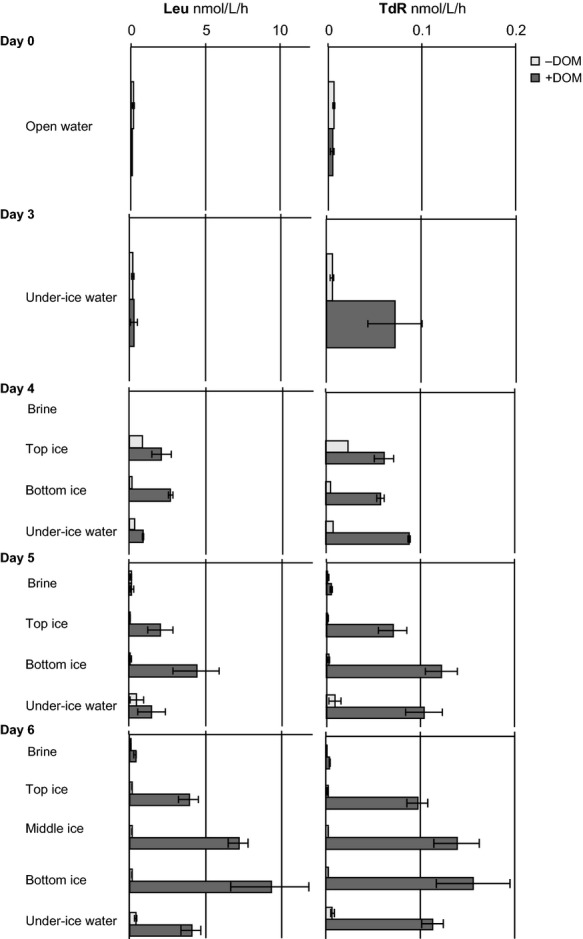
Total leucine (Leu) and thymidine (TdR) incorporation indicating bacterial protein and DNA synthesis in North Sea water and sea-ice experimental units. −DOM refers to unenriched and +DOM-enriched samples. Bars denote standard deviation between experimental units sampled on the respective sampling occasion. Note the different scale used in the two panels.

Salinity-normalized bacterial production measurements are presented in Figure 2. In the waters, the total TdR incorporation (a measure of bacterial DNA synthesis) was significantly higher in the DOM-enriched samples than in the unenriched samples (*W* = 338, *P* = 0.02046). In the total Leu incorporation (an indicative measure for bacterial protein synthesis), the signed-rank test used did not identify significant differences between the unenriched and DOM-enriched samples. In the unenriched water samples, both Leu and TdR increased only slightly from their initial values and reached maxima on the second ice-sampling day (5). In the DOM-enriched under ice water samples, there was a pronounced increase in TdR immediately after freezing began (day 3) that continued until the end of the experiment, whereas an increase in Leu was not detected until day 4.

In the ice, the temporal succession of Leu and TdR uptake incorporation rates reflected those in the water. For ice, both rates were significantly higher in the DOM-enriched samples (*W* = 306, *P* = 4.408 × 10^−10^ for both). In brines, the Leu and TdR incorporation rates were low compared with those measured in the bulk sea ice.

### Bacterial community succession

**Figure 3:**
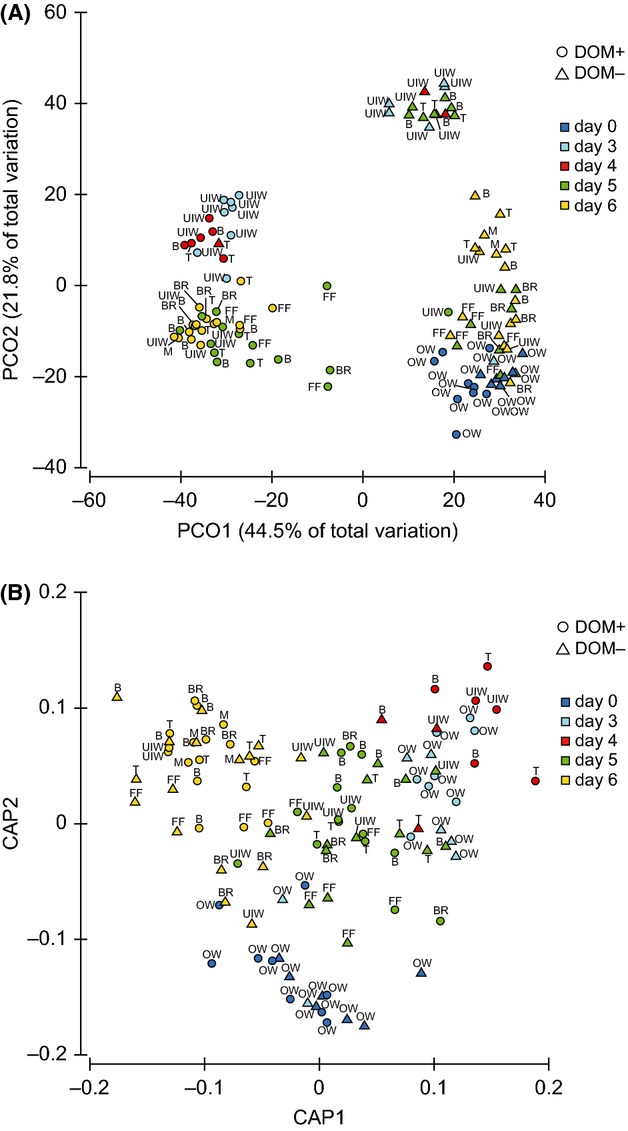
Changes in bacterial communities in unenriched and DOM-enriched North Sea water and sea-ice experimental units. (A) Principal coordinate (PCO) analysis of MspI-digested 16S rRNA gene terminal restriction fragments (T-RFs). In total, PCO axes 1 and 2 derived from Bray–Curtis distances explained 66.3% of original variation in the T-RF data. (B) Generalized discriminant analysis plots of MspI-digested 16S rRNA gene T-RFs. −DOM refers to unenriched and +DOM-enriched samples. t = top ice, b = bottom ice, br = brine, ff = frost flowers, uiw = under ice water and ow = open water.

Generalized discriminant analysis (Anderson and Robinson 2003) showed significant differences in T-RFs between the unenriched and DOM-enriched samples, as well as over time (*P* = 0.0001 with 9999 permutations for both) with all three restriction enzymes (Fig. 3B). Only the results obtained with MspI are shown, as it yielded the best separation of bacterial taxa at the class level. Between treatments, the first nine axes (choice of *m* = 9) gave the smallest cross-validation error (misclassification 5.8%) and explained 90.3% of the total variability in the T-RF data. Over time the corresponding values were *m* =14, misclassification 21.2%, and 96.3% explained.

In addition to discriminant analysis, PCO analysis showed there to be a clear separation between bacterial communities in the unenriched and DOM-enriched samples that illustrates the direct effect of DOM-enrichment on the bacterial communities (Fig. 3A). The most pronounced change occurred after the initiation of freezing (days 3 and 4). The unenriched and DOM-enriched samples fell into two separate groups, except the initial DOM-enriched water samples (day 0) that clustered together with the initial unenriched water samples (Fig. 3A). Clustering of the initial seawater communities was also observed in the discriminant analysis (Fig. 3B).

**Figure 4:**
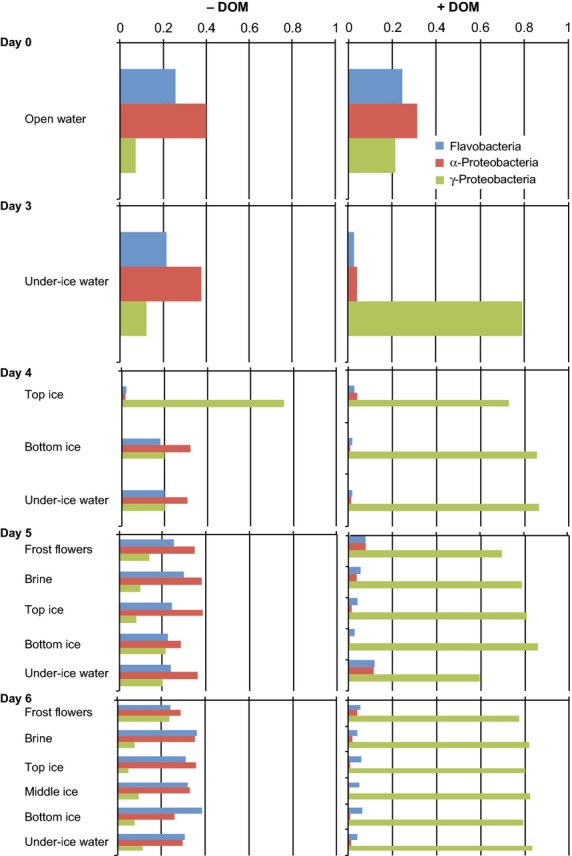
Bacterial succession between most abundant bacterial classes in North Sea water and sea-ice experimental units. −DOM refers to unenriched and +DOM-enriched samples. Determination of bacteria is based on relative fluorescent intensities of most abundant and identified MspI-digested 16S rRNA gene terminal restriction fragments (T-RFs). *X*-axis presents relative fluorecent intensities of terminal restriction fragment (T-RF) peak areas.

Based on the most abundant T-RFs identified (Table 2), *γ*-Proteobacteria dominated the community in the DOM-enriched samples throughout the experiment, except the initial water samples (Fig. 4). In contrast to the DOM-enriched samples, the *γ*-Proteobacteria dominated only briefly in the top ice layer of the unenriched ice (day 4), after which *α*-Proteobacteria began to dominate alongside Flavobacteria (Fig. 4). Brine, frost flower, and under ice water samples followed more or less the same patterns as the sea-ice samples in both the unenriched and DOM-enriched samples (Fig. 4).

**Table 2:** The most abundant expected and observed in silico and in vitro MspI-digested 16S rRNA gene terminal restriction fragments (T-RFs) derived from North Sea water and sea-ice experimental units.

Accession number	Expected T-RF in silico MspI (bp)	Observed T-RF in vitro MspI (bp)	Lowest rank^1^
**HE979573**	436	435	*Roseovarius* (genus)
**HE979595**	436	436	*Rhodobacteraceae* (family)
HE979638	438	435, 438	*Rhodobacteraceae* (family)
**HE979591**	438	439	*Thalassobacter* (genus)
**HE979600**	438	437	*Rhodobacteraceae* (family)
**HE979584**	438	438	*Phaeobacter* (genus)
**HE979585**	438	438	*Phaeobacter* (genus)
**HE979581**	440	440	*Rhodobacteraceae* (family)
**HE979596**	487	488, 490	*Glaciecola* (genus)
**HE979599**	489	491	*Pseudomonas* (genus)
**HE979597**	495	499	*Colwellia* (genus)
HE979626	493	494, 496	*Colwellia* (genus)
HE979657	493	495	*Shewanella* (genus)
HE979661	494	495	*Shewanella* (genus)
HE979641	495	495, 497	*Colwellia* (genus)
HE979659	495	496, 478	*Shewanella* (genus)
HE979623	495	476, 498	*Shewanella* (genus)
**HE979613**	88	83, 85	*Tenacibaculum* (genus)
**HE979586**	90	87	*Olleya* (genus)
**HE979586**	90	85, 87	*Olleya* (genus)
**HE979577**	90	85, 87	*Tenacibaculum* (genus)
**HE979578**	90	85, 87	*Polaribacter* (genus)
**HE979578**	90	85, 87	*Polaribacter* (genus)
**HE979564**	92	87, 89	*Flavobacteriaceae* (family)
HE979635	92	87, 89	*Flavobacteriaceae* (family)
HE979570	92	87, 89	*Flavobacteriaceae* (family)
HE979614	94	89, 91	*Tenacibaculum* (genus)
HE979568	94	89, 91	*Tenacibaculum* (genus)

bp = base pair; normal font = T-RF derived from initial unenriched water sample; bold = T-RF derived from unenriched bottom ice sample; underlined = T-RF derived from DOM-enriched bottom ice sample.

1 Results obtained with Ribosomal Database Project (RDP, Wang et al. 2007) naïve Bayesian rRNA classifier (v. 2.4, RDP training set 7), with 80% confidence threshold level.

### Bacterial community composition, richness and evenness

**Figure 5:**
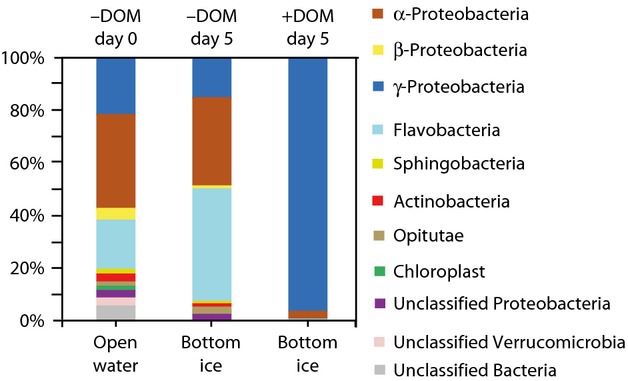
Class-level diversity of 16s rRNA gene clones in North Sea water and sea-ice experimental units. −DOM refers to unenriched and +DOM-enriched samples. Taxonomic classification was carried out using the Ribosomal Database Project (RDP) naïve Bayesian rRNA classifier (Version 2.4, Wang et al. 2007) with an 80% confidence threshold level.

*α*-Proteobacteria, *γ*-Proteobacteria, and Flavobacteria predominated in all three clone libraries constructed, but in different proportions (Fig. 5 and Table 3). Interestingly, new sea-ice-associated bacterial genera, including *Colwellia* sp., *Glaciecola* sp., and *Polaribacter* sp., appeared in the ice that were not detected in the initial seawater community. However, few of these sequences were found (Table 3).

**Table 3:** Number of bacterial genera in 16s rRNA gene clone libraries in North Sea water and sea-ice experimental units.

Class and lowest rank^1^	Unenriched open water (day 0)	Unenriched bottom ice (day 5)	DOM-enriched bottom ice (day 5)
Gammaproteobacteria			
*** Marinobacter***	1	ND	ND
*** Shewanella***	8	1	73
*** Halomonas***	1	ND	ND
* Alcanivorax*	1	ND	ND
*** Pseudomonas***	ND	2	ND
*** Glaciecola***	ND	2	ND
*** Colwellia***	ND	3	6
Unclassified Oceanospirillales	ND	1	ND
Unclassified *Alteromonadaceae*	ND	1	ND
Unclassified *Gammaproteobacteria*	3	2	1
Alphaproteobacteria			
*** Loktanella***	1	2	ND
* Roseovarius*	2	1	ND
* Thalassobacter*	ND	1	ND
* Phaeobacter*	ND	2	ND
** Unclassified *Rhodobacteraceae***	20	19	2
Betaproteobacteria			
* Methylotenera*	1	1	ND
Unclassified Betaproteobacteria	1	ND	ND
Unclassified Proteobacteria	2	2	ND
Flavobacteria			
* Gramella*	1	ND	ND
* Tenacibaculum*	2	11	ND
* Olleya*	2	7	ND
** Polaribacter**	ND	2	ND
Unclassified *Flavobacteriaceae*	7	12	1
Sphingobacteria			
* Haliscomenobacter*	1	1	ND
Actinobacteria			
* Ilumatobacter*	3	1	ND
Unclassified Actinomycetales	1	ND	ND
Opitutae			
* Puniceicoccus*	ND	1	ND
Unclassified *Puniceicoccaceae*	1	1	ND
Other			
Unclassified Verrucomicrobia	1	ND	ND
Chloroplast	2	ND	ND
Unclassified bacteria	3	ND	ND
Number of clones	65	76	83

Bold = sea ice bacteria; underlined = Baltic Sea ice bacteria; ND = not detected.

1 Taxonomic classification was carried out, using Ribosomal Database Project (RDP) naïve Bayesian rRNA classifier (V. 2.4, Wang et al. 2007), with 80% confidence threshold level.

**Figure 6:**
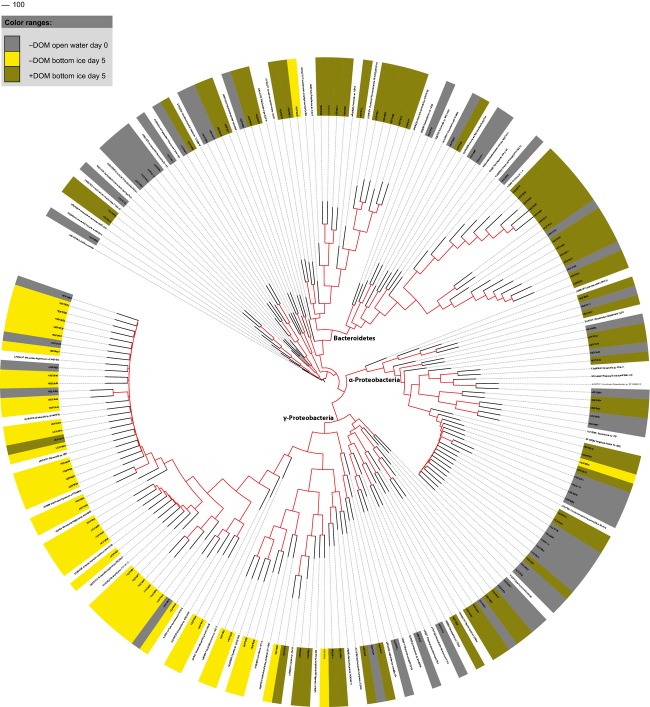
Phylogenetic neighbor-joining tree of 16S rRNA genes derived from North Sea water and sea-ice experimental units. Bootstrap values greater than 50% are shown with red. The length of the 16S rRNA was ˜950 bp. Sequence from archaeon *Sulfolobus tokodaii* (AB022438) was used as an outgroup in the alignment.

The bacterial communities differed significantly between the initial unenriched water and DOM-enriched bottom ice communities (*P* < 0.0001) as well as between unenriched bottom ice and DOM-enriched bottom ice (*P* < 0.0001). These differences suggest that DOM-enrichment induced the strongest changes in the bacterial communities. Moreover, the DOM-enriched bottom ice communities were separated into a distinct group from intermixed sequences of initial unenriched seawater and unenriched bottom ice communities in the phylogenetic NJ tree (Fig. 6).

*Shewanella* spp. that were already present in the initial unenriched water (Table 3 Fig. 6), dominated the community in the DOM-enriched samples representing 88% of all bacterial species present, with *Colwellia* sp. being the second most common species (Table 3). The overwhelming dominance of *γ*-Proteobacteria suggests that their response to the added DOM was superior to that of the other bacterial classes. In contrast to the DOM-enriched samples, Flavobacteria dominated the community together with *α*-Proteobacteria in the unenriched bottom ice (Table 3, Fig. 5).

The OTU numbers observed in the clone libraries began to reach saturation at an evolutionary distance of 0.15, except in the initial unenriched water sample (Figure S1). Both species richness and evenness were reduced after freezing, with the indices being lowest in the DOM-enriched samples (Table 4). As the same bacterial classes were almost equally abundant in clone libraries from the initial unenriched seawater and unenriched bottom ice, species evenness did not vary widely between them (Fig. 6, Table 4). However, due to the *γ*-Proteobacterial dominance in the DOM-enriched bottom ice (day 5), species evenness between the unenriched and DOM-enriched bottom ice was notably reduced.

**Table 4:** Richness and evenness indices for operational taxonomic units (OTU) at an evolutionary distance of 0.15 for the bacterial communities from North Sea water and ice experimental units.

Sample name	Number of sequences	Number of unique sequences	Number of OTUs	Chao1 richness	Simpson's evenness 1/D
Unenriched water (day 0)	65	58	16	38.5 (21.2–112.8^1^)	4.6
Unenriched bottom ice (day 5)	76	61	9	10 (9.1–19.7^1^)	3.2
DOM-enriched bottom ice (day 5)	83	46	3	3 (3–3^1^)	1.1

1 Indicates 95% confidence interval.

## Discussion

In both unenriched and DOM-enriched North Sea water treatments, the bacteria abundance in newly formed sea ice was higher than in initial water samples based on the enrichment index. Salinity-normalized bacterial abundance was highest in the frost flowers, exceeding that in the sea ice and brine, as was also found in previous study (Bowman and Deming 2010). It is generally known that bacteria become enriched (i.e., occur in excess compared to salinity) in sea ice, but usually the enrichment has been associated with algae (Grossmann and Gleitz 1993; Grossmann 1994; Grossmann and Dieckmann 1994; Helmke and Weyland 1995; Weissenberger and Grossmann 1998; Riedel et al. 2007b). In our experiment, the abundance of algae was extremely low (the experiment was conducted in darkness and the cell numbers were verified by epifluorescence microscopy, see Aslam et al. 2012), and thus are not likely to have played a role in the enrichment observed.

Bacterial production rates after sea-ice formation were significantly higher in the DOM-enriched samples than in the unenriched samples. In the DOM-enriched samples, bacterial production was already high on the first ice-sampling day and continued to increase throughout the experiment. In addition to the high level of bacterial production, high bacterial abundance (especially the HNA population), and decrease in DOC together with an increase in ammonium (NH_4_^+^) and particulate organic carbon (POC, see results in Aslam et al. 2012) indicated an active use of added substrate for metabolism in the DOM-enriched samples. Additionally, the decrease in O_2_ in the under ice water in the DOM-enriched samples indicated high bacterial respiration rates and thus the use of added substrate (see results in Aslam et al. 2012; Kirchman 2012). Bacterial production in the unenriched samples decreased after the second ice-sampling day, unlike in the DOM-enriched sea-ice samples. Moreover, the POC and DOC concentrations, as well as the bacterial abundance, decreased (see results in Aslam et al. 2012), indicating that there was suppression in bacterial growth.

Bacterial production rates in the brines did not follow the trends of those measured in ice. Despite the high bacterial abundance in brine, bacterial production was low in both unenriched and DOM-enriched brines. This can be explained by the brine-sampling technique in which partitioning of brines may occur and the more active bacteria are retained in the sea ice instead of the brine fraction (Becquevort et al. 2009; Kaartokallio et al. 2013).

As for the bacterial production measurements, clear differences between the unenriched and DOM-enriched samples were also seen at the bacterial community level. Both DOM treatment and time resulted in significant changes to the communities. The bacterial communities diverged immediately after freezing began in both treatments. These changes in the communities were most likely driven by ice formation, concentrating the DOM and nutrients in the brines, and presumably favoring a selection of psychrotrophic or psychrophilic bacteria (Helmke and Weyland 1995).

Significant changes in the bacterial community composition were seen between the unenriched and DOM-enriched samples. In all sample types, *α*-Proteobacteria, *γ*-Proteobacteria, and Flavobacteria were the most abundant classes found. The same bacterial classes have been shown to be present in natural sea ice, in different proportions, depending on the sampling time, location, and sample type (Brown and Bowman 2001; Junge et al. 2002; Brinkmeyer et al. 2003; Kaartokallio et al. 2008; Bowman et al. 2012). Even the bacterial communities in the frost flowers were very similar to those in the underlying sea ice, in contrast to previously described Arctic frost flower communities (Bowman et al. 2013). This is possibly because of the different geographical location of sampled seawater and because the frost flowers in this study grew without the influence of the surrounding natural environment.

In general, sea-ice communities in the unenriched samples reflected those of the seawater from which they were derived (this study: Collins et al. 2010), although the bacterial richness notably decreased and Flavobacteria dominated *α*-Proteobacteria in contrast to the initial water community. In addition, certain genera, such as *Colwellia*, *Glaciecola*, and *Polaribacter* that frequently inhabit sea ice (Deming 2010 and references therein) appeared only after sea-ice formation. The appearance of new taxa along with ice formation is consistent with the notion of the “rare biosphere” (Pedros-Alio 2006, 2012; Sogin et al. 2006), which suggests that as environmental conditions change, previously rare bacterial taxa will appear. Although the sequences were not detected in the initial water community, probably due to the low coverage of the clone library at the genus level, the result suggests that the relative abundance of *Colwellia* sp., *Glaciecola* sp., and *Polaribacter* sp. increased in ice as a result of the ice formation.

*γ*-Proteobacteria, more precisely the genera *Shewanella* and *Colwellia*, predominated in all sample types in the DOM-enriched samples throughout the experiment, except in the initial water community. These genera most likely contributed to the major proportion of the total bacterial abundance (HNA population) and DOC consumption in these samples. Both *Shewanella* sp. and *Colwellia* sp. have been found in natural sea ice (Bowman et al. 1997; Brown and Bowman 2001; Junge et al. 2002; Brinkmeyer et al. 2003). *γ*-Proteobacteria have been described as being abundant in spring/summer sea ice (Brinkmeyer et al. 2003; Kaartokallio et al. 2008) when there is labile DOM available for the bacteria following the spring ice algal blooms. *γ*-Proteobacteria have been reported to also dominated in Arctic multiyear ice in (Bowman et al. 2012).

In the unenriched samples, however, *γ*-Proteobacteria predominated only temporarily in the uppermost ice layer on the first ice-sampling day. This fleeting dominance of *γ*-Proteobacteria was probably due to the four times lower substrate levels (limiting growth) in the initial water of the unenriched samples compared to the DOM-enriched samples. The sudden decrease in *γ*-Proteobacteria in the unenriched samples could also have been due to viral attack, as very high virus-to-bacteria ratios have been reported in Arctic sea ice (Collins and Deming 2011). Grazing effects can be excluded, as there were very low numbers of heterotrophic flagellates present (see Aslam et al. 2012). Unfortunately, due to the two-thirds loss of replicates on day 4, the transient predominance of *γ*-Proteobacteria in the unenriched mesocosms is only hypothetical.

With the exception of the transient *γ*-Proteobacterial occurrence, Flavobacteria and *α*-Proteobacteria predominated in the unenriched samples throughout the experiment. Certain *γ*-Proteobacteria and *α*-Proteobacteria (family Rhodobacteraceae), benefit from high concentrations of LMW compounds (Teira et al. 2010; Alonso-Saez et al. 2012), implying that these bacteria in our study had similar substrate preferences. The dominance of *γ*-Proteobacteria suggests that they can effectively outcompete *α*-Proteobacteria under high nutrient concentrations, as previously suggested by Pinhassi and Berman (2003). Similar to open-water communities (Eilers et al. 2000; Fuchs et al. 2000; Pinhassi and Berman 2003; Allers et al. 2007; Teira et al. 2008, 2010; Gomez-Consarnau et al. 2012), the opportunistic growth of *γ*-Proteobacteria also seems possible in sea-ice under elevated concentrations of labile substrates. Conversely, the increase in Flavobacteria in the unenriched samples suggests their superior competitiveness over *α*-and *γ*-Proteobacteria in sea ice under lower nutrient concentrations.

## Conclusions

An active sea-ice bacterial community, similar to those previously described in natural sea ice, developed in sea ice experimentally grown from temperate North Sea water that does not typically freeze and produce sea ice.

Bacterial abundances relative to salinity were notably higher in ice compared to initial seawater in both unenriched and DOM-enriched treatments despite the absence of algae and other protists. Bacterial production and the changes in bacterial communities were pronounced in the DOM-enriched experimental units, pointing to the importance of substrate supply as a regulator of bacterial growth in sea ice and underlying waters. The bacterial communities in the unenriched samples were not different from those in the initial seawater, whereas the bacterial communities in the DOM-enriched samples were significantly different from the communities in initial seawater and unenriched samples. The unenriched sea-ice communities were dominated by Flavobacteria, *α*-proteobacteria, and *γ*-proteobacteria, whereas the DOM-enriched community was taken over by *γ*-Proteobacteria, indicating their opportunistic growth due to the elevated concentrations of available DOM. In all, the results show that seawater bacterial communities have a capacity to rapidly adapt when facing considerable changes in temperature, salinity, or supply of nutrients and organic matter occurring e.g., during ice formation.

## Supporting Information

Figure S1 Rarefaction analysis of 16s rRNA gene clone library sequences from (A) unenriched North Sea water (day 0), (B) unenriched bottom ice (day 5), and (C) DOM-enriched bottom ice (day 5) samples.
