# Supplementary material for: Bacterial community dynamics and activity in relation to dissolved organic matter availability during sea-ice formation in a mesocosm experiment
Source: Microbiologyopen. 2014 Jan 20;3(1):139–56. doi: 10.1002/mbo3.157 (PMC3937737; doi:10.1002/mbo3.157)
Supplement: Figure S1 — Rarefaction analysis of 16s rRNA gene clone library sequences from (A) unenriched North Sea water (day 0), (B) unenriched bottom ice (day 5), and (C) DOM-enriched bottom ice (day 5) samples. [file mbo30003-0139-sd1.docx]

**Bacterial community dynamics and activity in relation to dissolved organic matter availability during sea-ice formation in a mesocosm experiment**

Eeva Eronen-Rasimus*, Hermanni Kaartokallio, Christina Lyra, Riitta Autio, Harri Kuosa, Gerhard S. Dieckmann and David N. Thomas

*Corresponding author

Finnish Environment Institute (SYKE), Marine Research Centre, Erik Palménin aukio 1, 00560 Helsinki, Finland

Tel. (+358) 40 182 31 72

**Figure S1.** Rarefaction analysis of 16s rRNA gene clone library sequences from A.) Unenriched North Sea water (day 0), B.) Unenriched bottom ice (day 5) and C.) DOM-enriched bottom ice (day 5) samples.

A.)

B.)

C.)
